# A New Fast Control Strategy of Terminal Sliding Mode with Nonlinear Extended State Observer for Voltage Source Inverter

**DOI:** 10.3390/s23083951

**Published:** 2023-04-13

**Authors:** Chunguang Zhang, Donglin Xu, Jun Ma, Huayue Chen

**Affiliations:** 1 School of Automation and Electrical Engineering, Dalian Jiaotong University, Dalian 116028, China; 2 Traction Power State Key Laboratory, Southwest Jiaotong University, Chengdu 610031, China; 3 School of Computer Science, China West Normal University, Nanchong 637002, China

**Keywords:** voltage source inverter, uncertainty, extended state observer, fast terminal sliding mode control

## Abstract

To overcome the sensitivity of voltage source inverters (VSIs) to parameter perturbations and their susceptibility to load variations, a fast terminal sliding mode control (FTSMC) method is proposed as the core and combined with an improved nonlinear extended state observer (NLESO) to resist aggregate system perturbations. Firstly, a mathematical model of the dynamics of a single-phase voltage type inverter is constructed using a state-space averaging approach. Secondly, an NLESO is designed to estimate the lumped uncertainty using the saturation properties of hyperbolic tangent functions. Finally, a sliding mode control method with a fast terminal attractor is proposed to improve the dynamic tracking of the system. It is shown that the NLESO guarantees convergence of the estimation error and effectively preserves the initial derivative peak. The FTSMC enables the output voltage with high tracking accuracy and low total harmonic distortion and enhances the anti-disturbance ability.

## 1. Introduction

In recent years, with the development and utilization of smart grid and new energy, the key technology of modern inverter technology has been paid more and more attention [1]. However, due to the influence of various time-varying perturbations such as DC bias, load change, and filter parameter perturbation, the inverter output voltage has different degrees of distortion [2,3,4,5]. Therefore, it is of great theoretical significance and engineering value to design a reasonable robust control strategy for the inverter system to make its output voltage have better steady-state and dynamic performance [6,7,8,9,10,11,12].

At present, there are four methods to study the anti-interference performance of inverter systems. The first is using an artificial intelligence control algorithm [13,14,15,16,17]. In [13], a radial basis function neural-network-based approach is proposed to extract harmonics and solve the power quality problem for multilevel inverters. In [14], a robust iterative learning control technique is proposed, which can effectively suppress the influence of periodic and aperiodic disturbances of voltage source inverters. In [15], the fuzzy neural network method is used to realize on-line compensation, but the rate of convergence is slow. In [16], the neural network method is used to eliminate the voltage deviation disturbance caused by primary control, so as to recover its voltage reference value, but it requires many parameters and takes too long to train. In [17], the nonconvex condition is transformed into a convex linear matrix inequality to regulate the power share among distributed power sources and the system load voltage, so as to achieve good control performance; the disadvantage is that it is difficult to carry out system stability analysis. The second method is robust control [18,19]. In [18], the H2/H∞ hybrid optimal control method is proposed to resist the internal parameter change and external load disturbance of the inverter system. To solve the stability of voltage source inverters with LC filters, an H∞ controller with explicit robustness to grid impedance variations is proposed [19]. The third method is nonlinear control [20,21,22]. In [20], a novel continuous control set model predictive control for an LC-filter, three-phase, three-wire voltage source inverter is proposed. Model predictive control can effectively improve the control delay and reduce the output current distortion, but it requires accurate computation of the mathematical model of the controlled system, which reduces the robustness of the system. In [21], the calculations required for model prediction are reduced, and the optimal voltage vector can be found by calculating only four voltage vectors in each cycle. In [22], a novel MPC method using joint voltage vectors is proposed. The proposed scheme reduces the inductance current ripple and output current error of the inverter and simplifies the weight coefficient design in model predictive control. In [23], a backstepping sliding mode control (SMC) strategy is proposed for single-phase voltage source inverters, but in the design of backstepping controllers for higher-order systems, the need to derive the intermediate virtual control quantities one at a time leads to a “computational explosion” problem. In [24], an output-impedance-shaping method based on adaptive sliding mode control is proposed for voltage regulation, load proportional sharing, and ripple optimization of a DC microgrid. The fourth is a control method based on a disturbance observer. In [25], a disturbance observer is used to estimate the load disturbance and combined with SMC to further enhance the robustness and tracking accuracy of the system. An extended state observer (ESO) can estimate the variables and disturbances of state in real time, and because of their excellent disturbance observation capability, grid-connected inverter systems are widely used to suppress filter parameter variations and grid disturbances in [26]. Traditional sliding mode control suffers from the inability to stop tracking errors; although this can be solved by using terminal sliding modes, there are singularity problems. In addition, there are inherent jitter problems with sliding mode control that must be suppressed. A combination of disturbance estimation can generally be used. It can be seen from the above analysis that although the main control methods proposed so far have their own advantages in the application of inverter systems, they still suffer from different degrees of imperfections, and there are still numerous problems to be solved [27,28,29,30,31,32]. For example, problems include the chattering and convergence problems of SMC as well as the ESO initial peak problem. Fast terminal sliding mode control ensures that the state of the system converges to the equilibrium point within a finite time during the sliding phase by constructing a nonlinear sliding mode surface [33,34,35,36,37,38,39]. The chattering problem can be reduced by combining the sliding mode control with the observer. At the same time, consider that the initial peak suppression and fast-tracking capabilities of the nonlinear ESO are stronger than those of the linear ESO. In addition, some new methods have also been proposed in recent years [40,41,42,43,44,45,46,47,48].

Based on the characteristics of the third and fourth methods mentioned above, a nonlinear ESO-based FTSMC strategy is presented in this paper. The effects of load variations and filter parameter perturbations on the system are synthesized into a lumped uncertainty, which is estimated by a nonlinear ESO based on hyperbolic tangent functions. In order to improve the response speed and robustness of the inverter system, the lumped uncertainty of the nonlinear ESO reconstruction is considered, and an SMC law with terminal attractors is designed. The designed nonlinear ESO-based FTSMC strategy enables stable and reliable operation of the inverter system. Its output voltage tracking accuracy is high, and the total harmonic distortion (THD) is small. Moreover, the combination of nonlinear ESO and FTSMC can enhance the system’s ability to restrain perturbations.

The contributions of this paper are summarized below. 

(1) A novel nonlinear ESO-based fast terminal sliding mode control (FTSMC) strategy is presented as the core.

(2) The state space averaging method is used to establish the dynamic mathematical model of a single-phase voltage type inverter.

(3) The saturation property of the hyperbolic tangent function is employed to design the NLESO to estimate the lumped uncertainty. 

(4) A new sliding mode control method with a fast terminal attractor is proposed to improve the dynamic tracking of the system.

## 2. Problem Formulation

**Figure 1:**
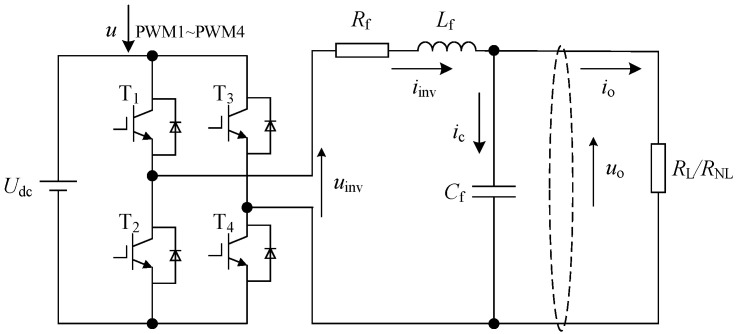
Block diagram of inverter system.

In this paper, the single-phase inverter system consists of a DC power supply, inverter, LC filter, and load, as shown in Figure 1.

In this figure, the power switch tube T_1_–T_4_ are IGBTs. The IGBTs are controlled by PWM, which converts DC power into AC power with adjustable fundamental frequency and amplitude. This is then transmitted to the load through a filter. $U_{\mathrm{dc}}$ is the DC voltage input to the inverter voltage. $i_{\mathrm{inv}}$ is the inductor current on the inverter side. $u_{\mathrm{inv}}$ and $u_{o}$ are the inverter output voltage and the filter output voltage, respectively. $L_{f}$, $C_{f}$, and $R_{f}$ are the filter inductors, capacitors, and resistors of the inverter, respectively. $Z_{L}/Z_{\mathrm{NL}}$ is the load disturbance, including linear and nonlinear loads.

The inverter system dynamic model is as follows:

(1) $$\left\{\begin{array}{l} L_{f}{\dot{i}}_{\mathrm{inv}}=u_{\mathrm{inv}}-u_{o}-R_{f}i_{\mathrm{inv}}\\ C_{f}{\dot{u}}_{o}=i_{\mathrm{inv}}-i_{o} \end{array}\right.$$

Rewrite Equation (1) as follows:

(2) $${\ddot{u}}_{o}=-\frac{R_{f}}{L_{f}}{\dot{u}}_{o}-\frac{1}{L_{f}C_{f}}u_{o}+\frac{uU_{\mathrm{dc}}}{L_{f}C_{f}}-\frac{1}{C_{f}}{\dot{i}}_{o}-\frac{R_{f}}{L_{f}C_{f}}i_{o}$$

Let us define the state variable as follows:

(3) $$\left\{\begin{array}{l} x_{1}=u_{o}\\ x_{2}={\dot{x}}_{1}={\dot{u}}_{o} \end{array}\right.$$

Considering the system internal parameter changes and external load disturbances, the system state equation is sorted as follows:

(4) $$\left\{\begin{array}{ll} {\dot{x}}_{1} & =x_{2}\\ {\dot{x}}_{2} & =(f(x_{1},x_{2})+\Delta f(x))+(b+\Delta b)u\;+D\\ & =f(x_{1},x_{2})+bu+d\\ y & =x_{1} \end{array}\right.$$

where $f(x_{1},x_{2})\;=\;-\frac{1}{L_{f}C_{f}}x_{1}-\frac{R_{f}}{L_{f}}x_{2}$; $\;b=\frac{U_{dc}}{L_{f}C_{f}}$; $D=-\frac{1}{C_{f}}{\dot{i}}_{o}-\frac{R_{f}}{L_{f}C_{f}}i_{o}$; d=D+Δf(x)+Δbu; D is external disturbance; and d is considered as complex time-varying disturbance. From the formula of d, the rth-order derivative of d is available.

In the physical world, the change rate of perturbation is always finite, which is the general case of perturbation estimation. That is to say, the rth-order derivative of d is bounded, satisfying $M=\underset{t\in (0,\infty )}{\sup}\left|d^{\left(r\right)}\right|<\infty$.

Thus, any unknown disturbance d can be represented by the Taylor expansion as follows:

(5) $$d=\sum_{i=0}^{r-1}a_{i}t^{i}+\xi (t)$$

where $a_{i}(i=0,1,\ldots ,r-1)$ are unknown constants, and ξ(t) is the residual term.

## 3. Controller Design and Stability Analysis

**Figure 2:**
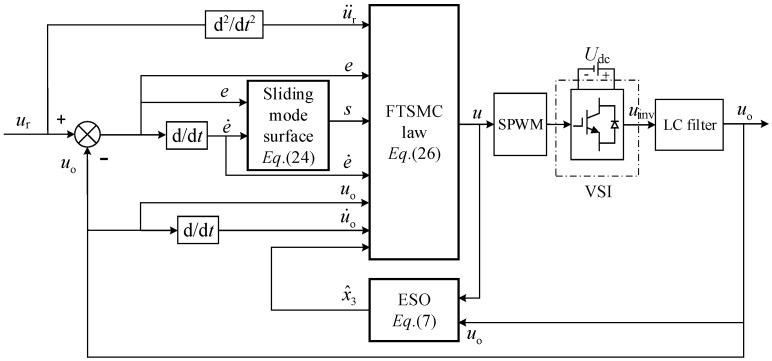
The block diagram of the proposed controller structure.

The control block diagram proposed is shown in Figure 2. Its core design includes nonlinear ESO and FTSMC. NLESO allows for better estimation of system disturbances and feedback compensation. In addition, the time-varying disturbance is considered; the FTSMC strategy with terminal attractor is used in here.

### 3.1. Nonlinear ESO Design Based on Hyperbolic Tangent Function

#### 3.1.1. Nonlinear ESO Design

Before the nonlinear ESO is designed, the unknown lumped uncertainty d is extended: $x_{3}=d$. Then we set $q(t)={\dot{x}}_{3}$, and $M=\underset{t\in (0,\infty )}{\sup}\left|q(t)\right|<\infty$ [49]. Therefore, Equation (4) is rewritten as follows:

(6) $$\left\{\begin{array}{l} {\dot{x}}_{1}=x_{2}\\ {\dot{x}}_{2}=f(x_{1},x_{2})+bu+x_{3}\\ {\dot{x}}_{3}=q(t)\\ y=x_{1} \end{array}\right.$$

The known information is utilized as much as possible; the nonlinear ESO design based on hyperbolic tangent function can be modified [50,51].

(7) $$\left\{\begin{array}{l} {\dot{\hat{x}}}_{1}={\hat{x}}_{2}+\beta_{1}(y-{\hat{x}}_{1})\\ {\dot{\hat{x}}}_{2}=f({\hat{x}}_{1},{\hat{x}}_{2})+bu+{\hat{x}}_{3}+\beta_{2}(y-{\hat{x}}_{1})\\ {\dot{\hat{x}}}_{3}=\beta_{3}\cdot \tanh(b(y-{\hat{x}}_{1})) \end{array}\right.$$

where ${\hat{x}}_{i}$ is the estimate value of $x_{i}$, $\beta_{i}\;>0\;(i=1,2,3)$ is observer gain, and tanh(.) is a hyperbolic tangent function, which is defined as

(8) $$\tanh(b\cdot x)=\frac{e^{bx}-e^{-bx}}{e^{bx}+e^{-bx}},\;b>0$$

where *b* is used to regulate the rate of change of the tanh(.).

**Figure 3:**
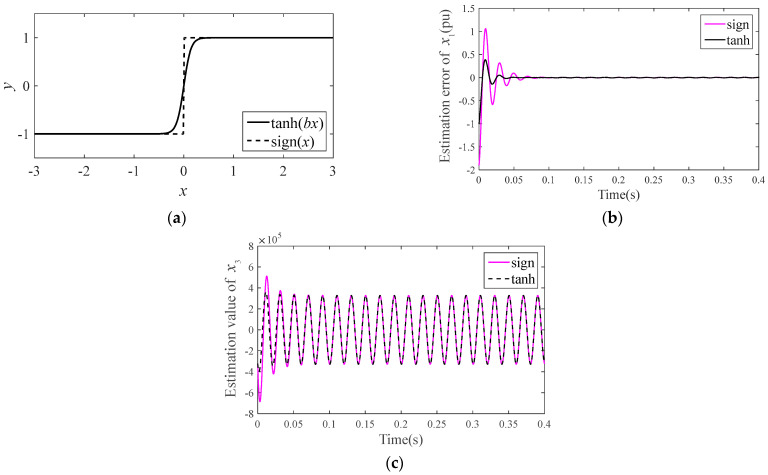
Effect comparison of ESO with different nonlinear functions: (**a**) comparison between hyperbolic tangent function and sign function; (**b**) the estimated error value on system state *x*_1_ of ESO with different nonlinear functions; and (**c**) the estimated value of extended state *x*_3_.

**Remark** **1.** *The sign function sign(.) is used in a conventional ESO, and the switch characteristic of sign(.) can easily cause high-frequency fluttering of the system and initial differential peak phenomenon. To solve this problem, the hyperbolic tangent function tanh(.) is used to replace sign(.). Figure 3 is a comparison of the tanh (.) and sign (.) functions.*

It can be seen that an ESO based on tanh(.) can effectively suppress the estimation peak value of a system state in the initial stage and obtain high estimation accuracy in other stages.

#### 3.1.2. Nonlinear ESO Convergence Analysis

**Assumption** **1.** f(.) is Lipschitz continuous. There exists a Lipschitz positive constant L, such that

(9) $$\left|f(x)-f(\hat{x})\right|\leq L\left\|x-\hat{x}\right\|,\;\forall \;x,\hat{x}\in R^{3}.$$

Let ${\tilde{x}}_{i}=x_{i}-{\hat{x}}_{i}\;(i=1,2,3)$ be the estimation error of ESO. In view of (6) and (7), it can obtained that

(10) $$\left\{\begin{array}{l} {\dot{\tilde{x}}}_{1}={\tilde{x}}_{2}-\beta_{1}{\tilde{x}}_{1}\\ {\dot{\tilde{x}}}_{2}={\tilde{x}}_{3}-\beta_{2}{\tilde{x}}_{1}+\delta (t)\\ {\dot{\tilde{x}}}_{3}=q(t)-\beta_{3}\cdot \tanh(b{\tilde{x}}_{1}) \end{array}\right.$$

where $\delta (t)=f(x_{1},x_{2})-f({\hat{x}}_{1},{\hat{x}}_{2})$.

For the hyperbolic tangent function, $\tanh(b\cdot {\tilde{x}}_{1})\approx b\cdot {\tilde{x}}_{1}$ in the neighborhood of ${\tilde{x}}_{1}=0$, so Equation (10) can be rewritten as

(11) $$\left\{\begin{array}{l} {\dot{\tilde{x}}}_{1}={\tilde{x}}_{2}-\beta_{1}{\tilde{x}}_{1}\\ {\dot{\tilde{x}}}_{2}={\tilde{x}}_{3}-\beta_{2}{\tilde{x}}_{1}+\delta (t)\\ {\dot{\tilde{x}}}_{3}=q(t)-b\beta_{3}{\tilde{x}}_{1} \end{array}\right.$$

Thus

(12) $$\dot{\tilde{x}}=A\tilde{x}+B\delta (t)+Cq(t)$$

where

$$\tilde{x}=\left[\begin{array}{l} {\tilde{x}}_{1}\\ {\tilde{x}}_{2}\\ {\tilde{x}}_{3} \end{array}\right],\;A=\left[\begin{array}{lll} -\beta_{1} & 1 & 0\\ -\beta_{2} & 0 & 1\\ -b\beta_{3} & 0 & 0 \end{array}\right],\;B=\left[\begin{array}{l} 0\\ 1\\ 0 \end{array}\right],\;C=\left[\begin{array}{l} 0\\ 0\\ 1 \end{array}\right].$$

Since A is a Hurwitz matrix, it satisfies the following conditions:

(13) $$A^{T}P+PA=-I.$$

Define the Lyapunov function as follows:

(14) $$V_{1}(\tilde{x})={\tilde{x}}^{T}P\tilde{x}.$$

It satisfies the following conditions:

(15) $$\lambda_{\min}(P){\left\|\tilde{x}\right\|}^{2}\leq V_{1}(\tilde{x})\leq \lambda_{\max}(P){\left\|\tilde{x}\right\|}^{2},$$

where $\lambda_{\min}(P),\;\lambda_{\max}(P)$ are the maximum eigenvalue and the minimum eigenvalue of the matrix P, respectively. $\left\|\;\cdot \;\right\|$ refers to the Euclid norm of $R^{3}$.

The derivative of $V_{1}(\tilde{x})$ is given by

(16) $$\begin{array}{l} \frac{dV_{1}(\tilde{x})}{dt}={\dot{\tilde{x}}}^{T}P\tilde{x}+{\tilde{x}}^{T}P\dot{\tilde{x}}\\ \;=\left[{\tilde{x}}^{T}A^{T}+B^{T}\delta (t)+C^{T}q(t)\right]P\tilde{x}\;\\ \;+{\tilde{x}}^{T}P\left[A\tilde{x}+B\delta (t)+Cq(t)\right]\\ \;=-{\left\|\tilde{x}\right\|}^{2}+2{\tilde{x}}^{T}PB\delta (t)+2{\tilde{x}}^{T}PCq(t)\\ \;\leq -{\left\|\tilde{x}\right\|}^{2}+2{\tilde{x}}^{T}PB\left|\delta (t)\right|+2{\tilde{x}}^{T}PCq(t) \end{array}$$

According to Cauchy–Schwarz inequality [52],

(17) $$\begin{array}{l} 2{\tilde{x}}^{T}PB\leq 2\left\|{\tilde{x}}^{T}\right\|\left\|P\right\|\left\|B\right\|=2\left\|{\tilde{x}}^{T}\right\|\left\|P\right\|\leq 2\lambda_{\max}(P)\left\|\tilde{x}\right\|\\ 2{\tilde{x}}^{T}PC\leq 2\left\|{\tilde{x}}^{T}\right\|\left\|P\right\|\left\|C\right\|=2\left\|{\tilde{x}}^{T}\right\|\left\|P\right\|\leq 2\lambda_{\max}(P)\left\|\tilde{x}\right\| \end{array}$$

In view of (9), (15) and (17), the (14) yields.

(18) $$\begin{array}{lll} \frac{dV_{1}(\tilde{x})}{dt} & \leq & -{\left\|\tilde{x}\right\|}^{2}+2\lambda_{\max}(P)L{\left\|\tilde{x}\right\|}^{2}+2\lambda_{\max}(P)\left\|\tilde{x}\right\|M\\ & \leq & -\frac{V_{1}(\tilde{x})}{\lambda_{\max}(P)}+\frac{2\lambda_{\max}(P)L}{\lambda_{\min}(P)}V_{1}(\tilde{x})\\ & & +\frac{2\lambda_{\max}(P)M}{\sqrt{\lambda_{\min}(P)}}\sqrt{V_{1}(\tilde{x})} \end{array}$$

It follows that

(19) $$\begin{array}{lll} \frac{d\sqrt{V_{1}(\tilde{x})}}{dt} & = & \frac{1}{2\sqrt{V_{1}(\tilde{x})}}\frac{dV_{1}(\tilde{x})}{dt}\\ & \leq & \frac{1}{2\sqrt{V_{1}(\tilde{x})}}[-\frac{V_{1}(\tilde{x})}{\lambda_{\max}(P)}+\frac{2\lambda_{\max}(P)L}{\lambda_{\min}(P)}V_{1}(\tilde{x})\\ & & +\frac{2\lambda_{\max}(P)M}{\sqrt{\lambda_{\min}(P)}}\sqrt{V_{1}(\tilde{x})}]\\ & = & (-\frac{1}{2\lambda_{\max}(P)}+\frac{\lambda_{\max}(P)L}{\lambda_{\min}(P)})\sqrt{V_{1}(\tilde{x})}+\frac{\lambda_{\max}(P)M}{\sqrt{\lambda_{\min}(P)}} \end{array}$$

We can obtain that

(20) $$\begin{array}{l} \sqrt{V_{1}(\tilde{x})}\leq \exp((-\frac{\lambda_{\min}(P)-2\lambda^{2}{}_{\max}(P)L}{2\lambda_{\max}(P)\lambda_{\min}(P)})(t-t_{0}))\sqrt{V_{1}(\tilde{x}(t_{0}))}\\ \;+\frac{\lambda_{\max}(P)M}{\sqrt{\lambda_{\min}(P)}}\int_{t_{0}}^{t}\exp((-\frac{\lambda_{\min}(P)-2\lambda^{2}{}_{\max}(P)L}{2\lambda_{\max}(P)\lambda_{\min}(P)})(t-\tau ))d\tau \end{array}$$

Combining (15) and (20) yields the following:

(21) $$\begin{array}{ll} \left\|\tilde{x}\right\| & \leq \frac{\sqrt{V_{1}(\tilde{x})}}{\sqrt{\lambda_{\min}(P)}}\leq \exp((-\frac{\lambda_{\min}(P)-2\lambda^{2}{}_{\max}(P)L}{2\lambda_{\max}(P)\lambda_{\min}(P)})\left(t-t_{0}\right))\sqrt{\frac{V_{1}(\tilde{x}(t_{0}))}{\lambda_{\min}(P)}}\\ & +\frac{\lambda_{\max}(P)M}{\lambda_{\min}(P)}\int_{t_{0}}^{t}\exp((-\frac{\lambda_{\min}(P)-2\lambda^{2}{}_{\max}(P)L}{2\lambda_{\max}(P)\lambda_{\min}(P)})(t-\tau ))d\tau \\ & \leq \exp((-\frac{\lambda_{\min}(P)-2\lambda^{2}{}_{\max}(P)L}{2\lambda_{\max}(P)\lambda_{\min}(P)})\left(t-t_{0}\right))\sqrt{\frac{\lambda_{\max}(P)}{\lambda_{\min}(P)}}\left\|\tilde{x}(t_{0})\right\|\\ & +\frac{2\lambda_{\max}(P)\lambda_{\min}(P)M}{2\lambda^{2}{}_{\max}(P)L-\lambda_{\min}(P)}[1-\exp((-\frac{\lambda_{\min}(P)-2\lambda^{2}{}_{\max}(P)L}{2\lambda_{\max}(P)\lambda_{\min}(P)})(t-t_{0}))] \end{array}$$

Finally, according to [21],

(22) $$\left\|{\tilde{x}}_{i}\right\|\leq \max\{\sqrt{\frac{\lambda_{\max}(P)}{\lambda_{\min}(P)}}\left\|\tilde{x}(t_{0})\right\|,\;\frac{2\lambda_{\max}(P)\lambda_{\min}(P)M}{2\lambda^{2}{}_{\max}(P)L-\lambda_{\min}(P)}\}$$

Thus, by choosing the observer gain properly, the estimation error of tanh(.)-based nonlinear ESO can converge to a small region.

### 3.2. Nonlinear ESO-Based FTSMC Design

#### 3.2.1. Nonlinear ESO-Based FTSMC Strategy

For uncertain systems (4), define the voltage output tracking error

(23) $$e=u_{r}-u_{o}.$$

In order to avoid the singularity of conventional terminal sliding mode, the non-singular fast terminal sliding mode surface is constructed [53,54,55].

(24) $$s=e+\frac{1}{\eta }e^{\left(g/h\right)}+\frac{1}{\mu }{\dot{e}}^{\left(p/q\right)}$$

where η>0,μ>0,g ,h ,p , q are positive constants, and 1<p/q<g/h<2.

Take the derivative of Equation (24):

(25) $$\begin{array}{lll} \dot{s} & = & \dot{e}+\frac{g}{\eta h}e^{(g/h)-1}\dot{e}+\frac{p}{\mu q}{\dot{e}}^{\left(p/q\right)-1}\ddot{e}\\ & = & \dot{e}(1+\frac{g}{\eta h}e^{(g/h)-1})+\frac{p}{\mu q}{\dot{e}}^{\left(p/q\right)-1}({\ddot{u}}_{r}-{\dot{x}}_{2})\\ & = & \dot{e}(1+\frac{g}{\eta h}e^{(g/h)-1})+\\ & & \frac{p}{\mu q}{\dot{e}}^{\left(p/q\right)-1}({\ddot{u}}_{r}-f(x_{1},x_{2})-bu-d) \end{array}$$

The sliding mode control input is designed as follows

(26) $$u=\frac{1}{b}\left(u_{1}+u_{2}\right),$$

where

(27) $$\left\{\begin{array}{ll} u_{1} & =(k_{1}s+k_{2}{\left|s\right|}^{\alpha }\mathrm{sign}(s))\\ & +\frac{\mu q}{p}{\dot{e}}^{2-(p/q)}(1+\frac{g}{\eta h}e^{(g/h)-1})+{\ddot{u}}_{r}\\ & -f(x_{1},x_{2})-{\hat{x}}_{3}\\ u_{2} & =\varphi \mathrm{sign}(s) \end{array}\right.$$

where $u_{2}$ is a robust control term to compensate the observation error of the ESO, and the φ value should meet the $\varphi >\left|{\hat{x}}_{3}-d\right|$. $k_{1}>0,k_{2}>0$, 0<α<1, and ${\hat{x}}_{3}$ is the estimated value of lumped uncertainty d of ESO of Equation (7).

#### 3.2.2. Stability Analysis of FTSMC

**Lemma** **1.** *Assume a continuous function* v(t) *satisfies the following differential inequality:*

(28) $$\dot{v}(t)\leq -a_{1}v(t)-b_{1}v{\left(t\right)}^{q_{1}/p_{1}},\;\forall t\geq t_{0}.$$

In addition, assume that

(29) $$t_{r}=t_{0}+\frac{p_{1}}{a_{1}(p_{1}-q_{1})}\ln(\frac{a_{1}v{\left(t_{0}\right)}^{\left(p_{1}-q_{1}\right)/p_{1}}+b_{1}}{b_{1}}),$$

where *a_1_* and *b_1_* are positive, $q_{1}$ and $p_{1}$ are positive odd constants, $0<q_{1}/p_{1}<1$, and $t_{0}$ is initial time. In that case, v(t) will converge to zero in finite time $t_{r}$.

Lyapunov function is chosen as $V_{2}=(1/2)s^{2}$, and the derivative of $V_{2}$ is

(30) $$\begin{array}{l} {\dot{V}}_{2}=s\frac{p}{\mu q}{\dot{e}}^{\left(p/q\right)-1}\left[\left({\hat{x}}_{3}-d)-k_{1}s-k_{2}{\left|s\right|}^{\alpha }\mathrm{sign}(s)-\varphi \mathrm{sign}(s\right)\right]\\ =\frac{p}{\mu q}{\dot{e}}^{\left(p/q\right)-1}[({\hat{x}}_{3}-d)s-k_{1}s^{2}-k_{2}{\left|s\right|}^{\alpha +1}-\varphi \left|s\right|]\\ \leq \frac{p}{\mu q}{\dot{e}}^{\left(p/q\right)-1}(-k_{1}s^{2}-k_{2}{\left|s\right|}^{\alpha +1}) \end{array}$$

Therefore, ${\dot{V}}_{2}\leq 0$, so according to Lyapunov stability theory, the control system is stable.

Then, Equation (29) can be further written as

(31) $${\dot{V}}_{2}\leq -\chi_{1}V_{2}-\chi_{2}V_{2}{}^{\left(\alpha +1\right)/2},$$

where $\chi_{1}=\frac{2k_{1}p}{\mu q}{\dot{e}}^{\left(p/q\right)-1},\chi_{2}=\frac{2^{\left(\alpha +1\right)/2}k_{2}p}{\mu q}{\dot{e}}^{\left(p/q\right)-1}$.

According to Lemma 1, the time for the sliding mode motion from $s(t_{0})\neq 0$ to converge to $s(T_{1})=0$ is

(32) $$T_{1}=t_{0}+\frac{1}{\chi_{1}(1-\left(\alpha +1\right)/2)}\ln\frac{\chi_{1}V_{2}{\left(t_{0}\right)}^{\left(1-\left(\alpha +1\right)/2\right)}+\chi_{2}}{\chi_{2}}$$

When the system enters the sliding surface $s(T_{1})=0$, according to Equation (24), the time can be obtained as follows:

(33) $$e+\frac{1}{\eta }e^{\left(g/h\right)}+\frac{1}{\mu }{\dot{e}}^{\left(p/q\right)}=0$$

Solving the error Equation (33), it can be obtained that the time after the system moves along the sliding mode surface to reach the zero-equilibrium state is set as follows:

(34) $$T_{2}=\frac{\mu \eta qhT_{1}+\eta hp\dot{e}{\left(T_{1}\right)}^{\frac{p}{q}-1}+\mu qge{\left(t_{0}\right)}^{\frac{g}{h}-1}T_{1}}{\mu \eta qh+\mu qge{\left(t_{0}\right)}^{\frac{g}{h}-1}}$$

Therefore, the motion state of the system will, from the initial state, converge to the zero equilibrium state in finite time $t_{f}=T_{1}+T_{2}$.

**Remark** **2.** *When the system state enters the sliding mode, Equation (33) has nothing to do with the lumped uncertainty d of the system. Therefore, the FTSMC strategy can guarantee the robustness of the system.*

**Remark** **3.** *When the system enters the sliding mode stage, that is s = 0, it can be obtained from Equation (33) that* $\frac{1}{\mu }{\dot{e}}^{\left(p/q\right)}$ *dominates when the sliding mode is far from the equilibrium, which guarantees a high convergence rate. In contrast, terminal attractor* $\frac{1}{\eta }e^{\left(g/h\right)}$ *dominates when the sliding mode is near the equilibrium, which guarantees a finite time convergence. The time of convergence can be obtained by solving the differential Equation (33). However, conventional sliding mode methods are not available. In addition, since* 1<p/q<g/h<2 *, the fast terminal sliding mode differential terms in Equation (25) avoid singularity.*

## 4. Simulation Analysis

To verify the performance of the FTSMC + NESO controller, the algorithm was modelled on the MATLAB/SIMULINK platform, and two more systems were built for comparison.

System 1 is the control strategy proposed in this paper (ESO + FTSMC). System 2 is a fast terminal sliding mode inverter control system without an ESO(FTSMC). System 3 is a conventional sliding mode inverter control system based on a nonlinear ESO(ESO + SMC).

In the simulation, the rated capacity of the inverter was set to 2.2 kVA, the inverter control system output reference voltage was $u_{r}=220\sqrt{2}\sin100\pi t$, and the circuit and control parameters were selected in the simulation as listed in Table 1 and Table 2.

### 4.1. Performance of System 1

**Figure 4:**
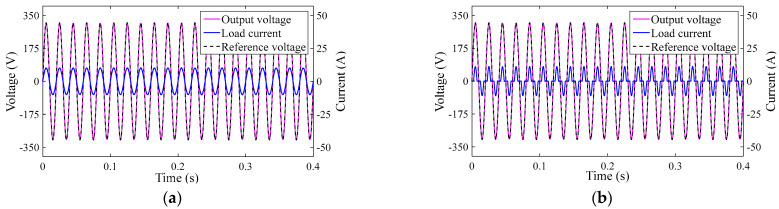
The output responses of System 1: (**a**) transient responses of System 1 in linear load and (**b**) transient responses of System 1 in nonlinear load.

The inverter output voltage and load current response for System 1 under two different load types are shown Figure 4.

It can be seen that the inverter output voltage can quickly track the reference input voltage under different loads. Figure 4a,b show that the inverter output voltage varies exactly in phase with the load current, but from Figure 4b, it can be seen that the inverter system output current is non-sinusoidal under nonlinear loads.

### 4.2. Comparative Study of System 1 and System 2

There is no nonlinear ESO in System 2. The design process of FTSMC and the control block diagram of the inverter system are shown in Appendix A. For the sake of the fairness and validity of the comparison, the corresponding FTSMC parameters and filter parameters were identical in System 1 and System 2.

#### 4.2.1. Comparison of System 1 and System 2 under Varying Linear Loads

**Figure 5:**
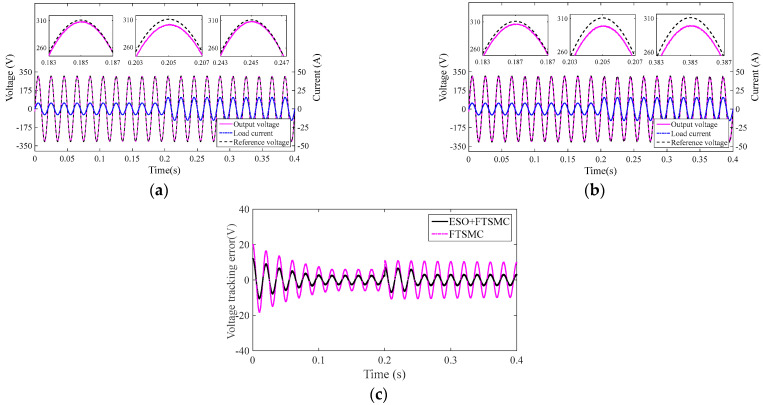
Inverter system output response under linear load saltation: (**a**) output response of System 1, (**b**) output response of System 2, and (**c**) voltage error.

The load values of System 1 and System 2 decreased from 38 Ω to 19 Ω at 0.2 s. Figure 5a,b are the output voltage and current output response of System 1 and System 2, respectively. Figure 5c is the voltage error between the output voltage of System 1 and System 2 and the reference voltage, respectively.

As can be seen from Figure 5a, when the load suddenly changes, the output voltage of System 1 deviates from the reference voltage, but after about two multi periods, it can track the reference voltage well. As shown in Figure 5b, when the load is suddenly changed, the output voltage of System 2 always deviates from its reference voltage. Figure 5c further shows that the output voltage of System 1 deviates from the reference voltage to a smaller degree than that of System 2.

#### 4.2.2. Comparison of System 1 and System 2 under Varying Nonlinear Loads

**Figure 6:**
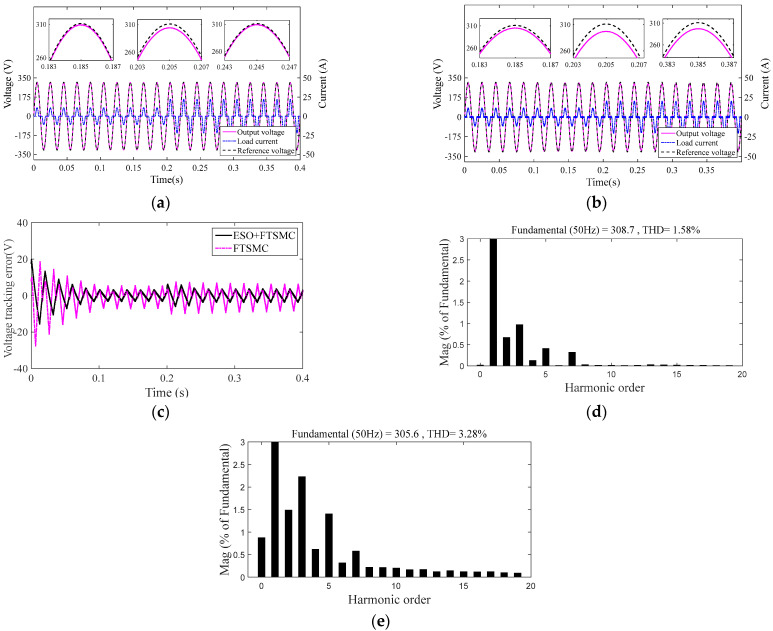
Inverter system output response under nonlinear load saltation: (**a**) output response of System 1, (**b**) output response of System 2, (**c**) voltage error, (**d**) output voltage THD of System 1, and (**e**) output voltage THD of System 2.

In this part, System 1 and System 2 parallel a set of the same nonlinear load at 0.2 s. Figure 6a,b are the output voltage and current output response of System 1 and System 2, respectively. Figure 6c shows the voltage error between the output voltage of System 1 and System 2 and the reference voltage, respectively. Figure 6d,e are the output voltage THD of System 1 and System 2.

It can be seen from Figure 6a, Figure 6b that when the nonlinear load suddenly changes, the output voltage of System 1 deviates from the reference voltage, but it tracks the reference voltage well after about two multi periods, while the output voltage of System 2 always deviates from its reference voltage. Figure 6c shows that the output voltage of System 2 deviates from its reference voltage to a greater extent than that of System 1. Figure 6d, Figure 6e further show that when the system suffers from nonlinear load saltation, the output voltage THD of System 1 and System 2 meet IEEE standard 519-2014 (THD < 5%), while System 1 has a smaller THD value. Therefore, System 1 with nonlinear ESO has a better ability to deal with nonlinear load disturbance than System 2.

#### 4.2.3. Comparison of System 1 and System 2 under Perturbation of Filter Parameters

**Figure 7:**
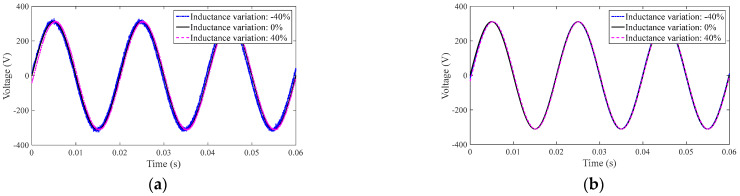
The output voltages of two voltage controllers: (**a**) output response of System 1 and (**b**) output response of System 2.

In this part, simulations are carried out for the case where the filter inductance value is 40% of the nominal parameter. Figure 7a,b show the output voltages of System 1 and System 2 for the three inductor parameter values, respectively.

As seen in Figure 7a, b, when the inductance parameter is ingested, the output voltage of System 1 is closer to that of System 2. System 1 with nonlinear ESO is therefore better able to accommodate inductance parameter ingress than System 2.

### 4.3. Comparative Study of System 1 and System 3

Conventional SMC is used in System 3. See Appendix A for its design process and inverter system control block diagram. System 1 and System 3 use the same nonlinear ESO, and the corresponding parameters and filter parameters are completely consistent.

**Figure 8:**
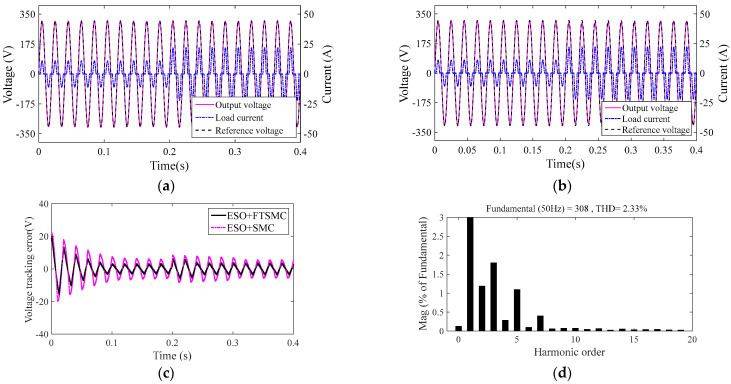
Inverter system output response under nonlinear load saltation: (**a**) output response of System 1, (**b**) output response of System 3, (**c**) voltage error, (**d**) output voltage THD of System 1.

System 1 and System 3 parallel a set of the same nonlinear load at 0.2 s. Figure 8a,b are the output voltage and current output response of System 1 and System 3, respectively. Figure 8c is the voltage error between the output voltage of System 1 and System 3 and the reference voltage, respectively. Figure 8d is the output voltage THD of System 1 and System 2, respectively.

As can be seen from Figure 8a,b, when the nonlinear load changes suddenly, the output voltage of System 1 deviates from the reference voltage, but it can track the reference voltage well after about two multi periods, while System 3 can only track the reference voltage after about five multi periods. Figure 8c shows that, compared with that of System 1, the output voltage of System 3 fluctuates greatly from its reference voltage. Figure 8d shows that when the system suffers from nonlinear load mutation, the output voltage THD of both System 1 and System 3 meets IEEE Standard 519-2014(THD < 5%), and System 1 has a smaller THD value. Since the terminal attractor introduced in the FTSMC strategy can promote the system, FTSMC-based System 1 has a faster response time than SMC-based System 3.

**Figure 9:**
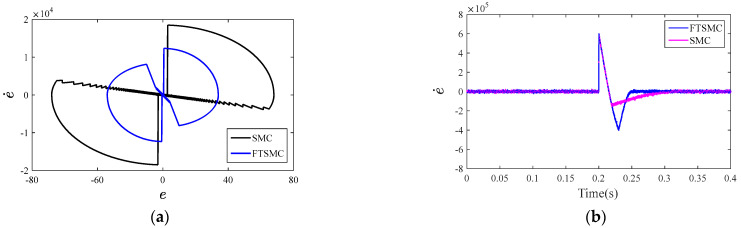
Simulated state trajectories: (**a**) the simulated state trajectories in the phase plane for the conventional SMC method and FTSMC method and (**b**) simulated responses of the rate of change of the output voltage error under the two methods.

The simulated state trajectories in the phase plane for the conventional SMC method and FTSMC method are shown in Figure 9a. Figure 9b shows the simulated responses of the rate of change of the output voltage error under the conventional SMC method and FTSMC method during the step change in the load current.

It can be seen from Figure 9a that the SMC method takes much more time to travel from the initial state to the equilibrium points than the FTSMC method. In addition, the responses obtained by the FTSMC method are much faster than those obtained by the SMC method, as shown in Figure 9b.

## 5. Conclusions

In this study, a nonlinear ESO-based FTSMC scheme for a VSI system is proposed. The proposed NESO-based FTSMC strategy can enable the inverter system to stably and reliably operate against disturbances such as load and filter parameter variations, providing satisfactory dynamic regulation and steady-state tracking of its output voltage to meet power quality requirements. The algorithm in this paper uses only voltage sensors and does not require current sensors, saving hardware costs. This paper provides a novel high-precision robust control method for distributed power-generation systems such as inverter-based systems. 

Due to the limitations of the conditions, only theoretical and simulation studies have been performed on the designed control strategy, and the next step will be experimental validation.

## Figures and Tables

**Table 1:** System electrical parameters [56].

Description	Parameters	Nominal Values
DC link voltage	$U_{\mathrm{dc}}$	400 V
Inverter switching	f	10 kHz
Parasitic resistance	$R_{f}$	0.2 Ω
Filter inductor	$L_{f}$	5 mH
Filter capacitor	$C_{f}$	10 μF
Linear load	$R_{L}$	38 Ω
Nonlinear load	$R_{\mathrm{NL}}$	(38 Ω+5 mH)∥2.5 mF

**Table 2:** System control parameters.

Parameters	Nominal Values
$\beta_{1},\beta_{2},\beta_{3}$	0.001, 0.04, 12
b	0.3
g,h,p,q	5, 3, 9, 7
η,μ	0.05, 0.02
$k_{1},k_{2},\varphi$	5, 1, 60
α	0.82

## Data Availability

All data, models, and code generated or used during the study appear in the submitted article.

## Appendix A

### Appendix A.1. The Design of System 2

Regarding Formula (24) of Section 3.2, as a sliding surface selection, in view of an inverter nominal system, the derivation of (24) is

(A1) $$\begin{array}{c} \dot{s}=\dot{e}(1+\frac{g}{\eta h}e^{(g/h)-1})+\frac{p}{\mu q}{\dot{e}}^{\left(p/q\right)-1}[{\ddot{u}}_{r}-f(x_{1},x_{1})\\ -bu+\frac{1}{C_{f}}{\dot{i}}_{o}+\frac{R_{f}}{L_{f}C_{f}}i_{o}] \end{array}$$

The input of sliding mode control is designed as

(A2) $$\begin{array}{ll} u= & \frac{1}{b}[k_{1}s+k_{2}{\left|s\right|}^{\alpha }\mathrm{sign}(s)+\frac{\mu q}{p}{\dot{e}}^{2-(p/q)}(1+\frac{g}{\eta h}e^{(g/h)-1})\\ & +{\ddot{u}}_{r}-f(x_{1},x_{2})+\frac{1}{C_{f}}{\dot{i}}_{o}+\frac{R_{f}}{L_{f}C_{f}}i_{o}] \end{array}$$

### Appendix A.2. The Design of System 3

The sliding surface of conventional sliding mode control is selected as

(A3) $$s=\dot{e}+ce.$$

The reaching law is designed as

(A4) $$\dot{s}=-k_{1}\mathrm{sign}(s).$$

For the uncertain inverter system (4), the derivation of (36) is

(A5) $$\dot{s}={\ddot{u}}_{r}-f(x_{1},x_{2})-bu-d+c({\dot{u}}_{r}-x_{2}).$$

The input of sliding mode control is designed as

(A6) $$u=\frac{1}{b}[{\ddot{u}}_{r}-f(x_{1},x_{2})-{\hat{x}}_{3}+c({\dot{u}}_{r}-x_{2})+k_{1}\mathrm{sign}(s)],$$

where c=20.
